# Ulcerated necrobiosis lipoidica: clinical and histopathological presentation of two cases^؆^

**DOI:** 10.1016/j.abd.2025.501279

**Published:** 2026-01-19

**Authors:** Vitória Mariah Giriboni, Lucas Silva Cortes, Simone Antunes Terra, Luciana Patricia Fernandes Abbade

**Affiliations:** Department of Infectology, Dermatology, Imaging Diagnosis and Radiotherapy, Faculty of Medicine, Universidade Estadual Paulista, Botucatu, SP, Brazil

Dear Editor,

Necrobiosis lipoidica (NL) is a rare granulomatous disease that affects more women than men. Its etiopathogenesis remains unexplained. Although it is most commonly associated with insulin-dependent diabetes, NL can coexist with thyroid dysfunction, sarcoidosis, rheumatoid arthritis, and metabolic syndrome. It presents as circumscribed, atrophic, yellowish-brown plaques with telangiectasias and violaceous borders, usually located in the pretibial region. About one-third of cases may progress to ulcerations, often resulting from minor trauma.^1^, ^2^

The present report describes two cases of ulcerated NL to demonstrate the diagnostic and therapeutic challenges. The first case is a 70-year-old woman with type 2 diabetes, good glycemic control, and non-insulin-dependent, with high blood pressure and heart disease. She was followed for 28 years in the dermatology outpatient clinic, due to NL in the lower limbs with the formation of recurrent ulcers, initially diagnosed as venous ulcers (Fig. 1). Doppler ultrasound showed no pathological changes in the deep and superficial venous system. Histopathological examination showed changes characteristic of NL (Fig. 2A and B). Superficial surgical debridement, anti-stasis measures, dressings for exudative and colonized lesions (e.g., hydrofiber with silver), oral use of pentoxifylline, colchicine, gabapentin (for pain control), and sulfamethoxazole-trimethoprim (for control of recurrent infections) were performed. She progressively improved in healing after the start of this antibiotic with maintenance for three months, although active NL lesions were still present (Fig. 3).

**Fig. 1:**
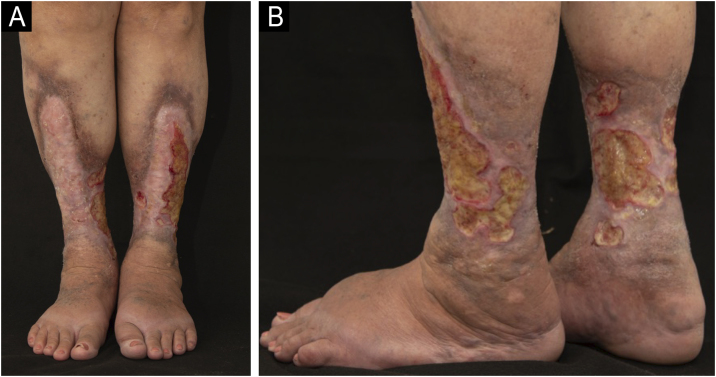
(A and B) Case 1. Ulcerated Necrobiosis lipoidica simulating venous ulcers. Ulcers are observed on the anterior, lateral, medial, and posterior aspects of the lower limbs, with fibrinoid tissue over an extensive atrophic plaque with a slightly yellowish center, brownish and purplish borders, affecting the entire distal circumference, associated with bilateral edema.

**Fig. 2:**
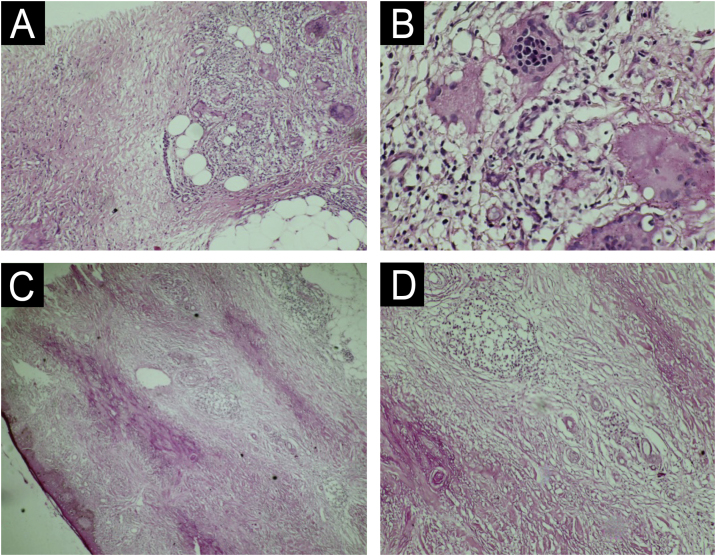
(A and B) Anatomopathological examination of Case 1 highlights multinucleated giant cells and the granulomatous arrangement around necrotic connective tissue (A – Hematoxylin & eosin, ×100; B – Magnified image; Hematoxylin & eosin, ×400). (C and D) Anatomopathological examination of Case 2 reveals alternating bands of necrotic connective tissue and areas of chronic inflammatory infiltrate in a palisade pattern, rich in lymphocytes, multinucleated giant cells, and plasma cells. Inflammatory cells are also distributed around appendages and vessels. The typical alternating architectural pattern resembles the layers of a cake (C – Hematoxylin & eosin, ×40; D – Hematoxylin & eosin, ×100).

**Fig. 3:**
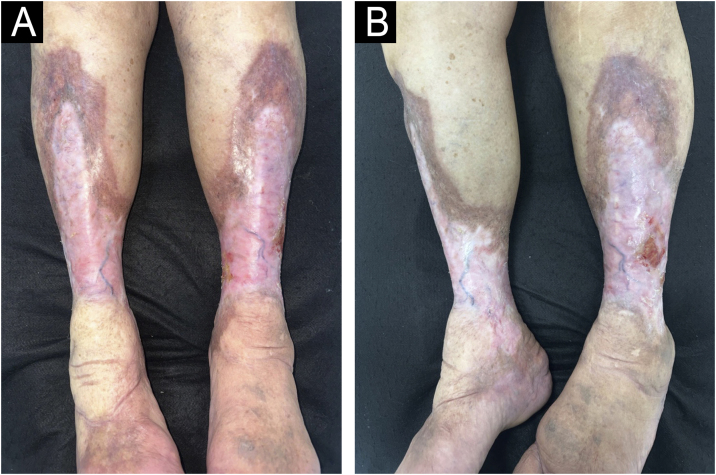
Case 1. Necrobiosis lipoidica with ulcers evolving towards healing. (A) Observe an extensive atrophic plaque with visualization of blood vessels, a slightly yellowish center, and brownish and purplish edges, affecting the entire distal circumference. (B) The single ulcerated lesion on the lateral aspect of the left leg is evident, superficial, and with good granulation tissue.

The second case is a 61-year-old woman, insulin-dependent diabetic, high bood pressure, dyslipidemic, with hypothyroidism, who underwent adrenalectomy due to adrenal carcinoma, being followed for five years in the dermatology outpatient clinic with the onset of hyperchromic plaques, with an atrophic and yellowish center, and well-defined and brownish borders in the pretibial region of the left lower limb associated with pruritus, with periods of ulceration and spontaneous healing (Fig. 4A). The histopathological examination was compatible with NL (Fig. 2C and D). She underwent surgical debridement, high-potency topical corticosteroid use when active lesions were present in the peri-ulcer skin, and anti-stasis measures, in addition to dressings depending on the healing stage. The lesions healed about a year ago without recurrence (Fig. 4B).

**Fig. 4:**
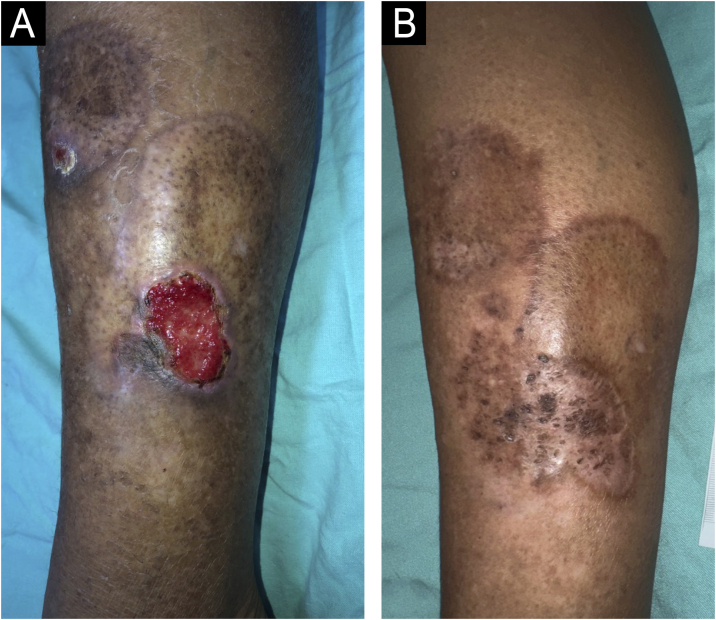
Case 2. (A) Chronic ulcer on the left lower limb over necrobiosis lipoidica. At this stage, a hydrocolloid dressing was used to maintain a moist environment and promote healing. (B) Healed ulcer.

NL requires attention and early recognition by dermatologists, endocrinologists, and clinicians in general, being a warning sign for diabetes screening in affected patients.^1^ Diagnosis requires the integration of clinical and histopathological findings. In histopathological examination, NL is characterized by granulomatous inflammation consisting of histiocytes and dispersed giant cells with dermal fibrosis and necrobiotic connective tissue, arranged in a “layer-cake” pattern. The blood vessels surrounding the necrobiotic connective tissue often show edema, fibrosis, and hyalinization of endothelial cells. The epidermis is usually not affected, but it may be atrophic or ulcerated.^3^

Ulcerated NL has differential diagnoses with other causes of chronic lower limb ulcers, mainly venous ulcers.^4^, ^5^ The NL plaque in the present case was initially confused with extensive lipodermatosclerosis; however, the patient did not have venous disease as evidenced by venous ultrasound, and the histopathology of the peri-ulcer lesion confirmed the diagnosis of NL. Late identification and delayed diagnosis can lead to disease progression, making it recalcitrant, particularly when ulceration occurs.^6^

NL treatment is challenging, and relapses occur frequently. Control of glycemic levels is recommended, although its relationship with disease control is controversial. The first line of treatment is topical and occlusive high-potency corticosteroids. In extensive and refractory cases, systemic corticosteroid therapy may be used, but evaluation of adverse events in diabetic patients is necessary. There are also reports of treatment with hydroxychloroquine, immunobiologicals (anti-Tumor Necrosis Factor-alpha – TNF-α), and Janus Kinase inhibitors (anti-JAK). According to the literature, about 20% of lesions show spontaneous resolution.^7^, ^8^, ^9^

In the case of ulcerated NL, the challenge is even greater. Treatment of possible secondary infections, serial debridement of devitalized tissues, compression therapy, and appropriate dressings are indicated. Anti-TNF-alpha and anti-JAK immunobiologicals represent promising therapeutic strategies for disease refractory to conventional treatments.^7^, ^8^, ^9^

To date, it remains a challenging condition since the evidence for treatments is based primarily on case reports, given the multiple therapeutic modalities available and the rarity of cases. It is of paramount importance to conduct controlled primary studies aimed at proposing a first-line treatment and thus avoid NL refractoriness. It is noted that chronic cases, such as the one reported, require constant monitoring due to the risk of malignant transformation, mainly to squamous cell carcinoma on areas of chronic ulceration.^10^

## ORCID IDs

Vitória Mariah Giriboni: 0000-0002-6124-1495

Lucas Silva Cortes: 0000-0002-2422-9665

Simone Antunes Terra: 0000-0001-7929-9014

## Authors' contributions

Vitória Mariah Giriboni: Design and planning of the study; drafting and editing of the manuscript; collection, analysis, and interpretation of data; intellectual participation in the propaedeutic and/or therapeutic conduct of the studied cases; critical review of the literature; critical review of the manuscript; approval of the final version of the manuscript.

Lucas Silva Côrtes: Drafting and editing of the manuscript; collection, analysis, and interpretation of data; intellectual participation in the propaedeutic and/or therapeutic conduct of the studied cases; critical review of the literature; critical review of the manuscript; approval of the final version of the manuscript.

Simone Antunes Terra: Drafting and editing of the manuscript; collection, analysis, and interpretation of data; intellectual participation in the propaedeutic and/or therapeutic conduct of the studied cases; critical review of the literature; critical review of the manuscript; approval of the final version of the manuscript.

Luciana Patrícia Fernandes Abbade: Design and planning of the study; drafting and editing of the manuscript; collection, analysis, and interpretation of data; intellectual participation in the propaedeutic and/or therapeutic conduct of the studied cases; critical review of the literature; critical review of the manuscript; approval of the final version of the manuscript.

## Financial support

None declared.

## Research data availability

Not applicable.

## Conflicts of interest

None declared.
